# Risk Assessment Considerations for Genetically Modified RNAi Plants: EFSA’s Activities and Perspective

**DOI:** 10.3389/fpls.2020.00445

**Published:** 2020-04-21

**Authors:** Nikoletta Papadopoulou, Yann Devos, Fernando Álvarez-Alfageme, Anna Lanzoni, Elisabeth Waigmann

**Affiliations:** Genetically Modified Organisms Unit, Department of Scientific Evaluation of Regulated Products Development, European Food Safety Authority, Parma, Italy

**Keywords:** crops, RNAi, dsRNA, DvSnf7, gene silencing, off-target, risk assessment, genetically modified organisms

## Abstract

Genetically modified plants (GMPs) intended for market release can be designed to induce “gene silencing” through RNA interference (RNAi). The European Food Safety Authority (EFSA) and other international risk assessment bodies/regulatory agencies have taken several actions to determine whether the existing risk assessment approaches for GMPs are appropriate for the risk assessment of RNAi-based GMPs or require complementary or alternative approaches. To our knowledge, at the international level, no dedicated guidelines have been developed for the risk assessment and regulation of RNAi-based GMPs, confirming that existing science-based risk assessment approaches for GMPs are generally considered suitable for RNAi-based GMPs. However, some specificities have been identified for the risk assessment of RNAi-based GMPs. Here, we report on some of these specificities as identified and addressed by the EFSA GMO Panel for the molecular characterisation, food/feed safety assessment and environmental risk assessment of RNAi-based GMPs, using the DvSnf7 dsRNA-expressing maize MON87411 as a case study.

## Introduction

**FIGURE 1:**
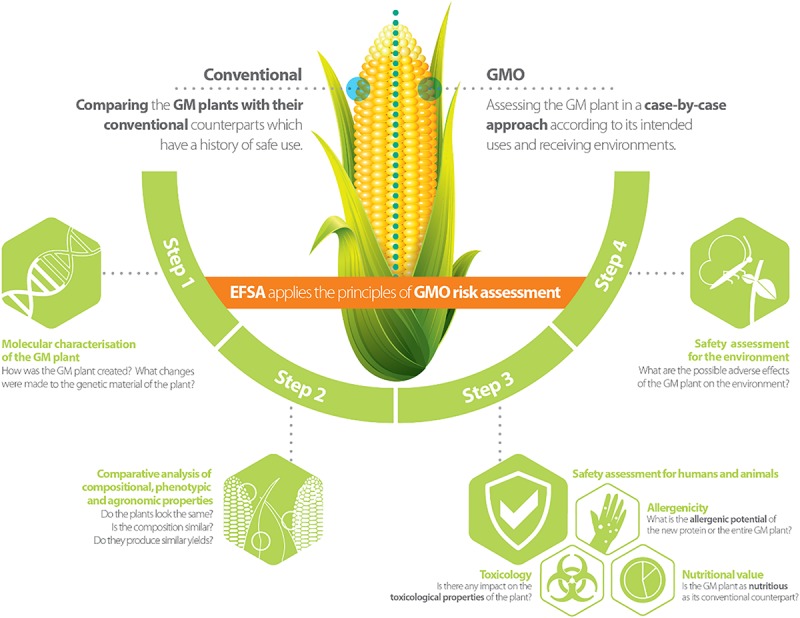
Risk assessment approach for genetically modified plants [reprinted with permission from EFSA’s infographic (Available at https://www.efsa.europa.eu/en/discover/infographics/risk-assessment-genetically-modified-plants; ISBN 978-92-9199-913-2 | doi: 10.2805/240762 | TM-02-17-009-EN-N)].

Genetically modified plants (GMPs) and/or derived food/feed (FF) products, are subject to a risk assessment and regulatory approval before entering the market in the European Union (EU). In this process, the role of the European Food Safety Authority (EFSA) is to assess and provide scientific advice to risk managers on any possible risks that the deployment (e.g., consumption or cultivation) of GMPs may pose to humans, animals and the environment (Waigmann et al., 2012). EFSA’s scientific advice on the risk assessment of GMPs is given through its scientific Panel on genetically modified organisms (GMOs) consisting of scientific experts coming from EU research institutes, universities or risk assessment bodies. For the evaluation of GMP market registration applications, EFSA’s GMO Panel is supported by the GMO Unit, and three standing working groups, each of which focuses on specific risk assessment areas addressing: (a) the molecular characterisation of GMPs; (b) the FF safety assessment of GMPs and/or derived FF products; and (c) the environmental risk assessment of GMPs (see Figure 1 for further details; Devos et al., 2014).

Plants can be engineered to induce gene silencing through RNA interference (RNAi). At present, RNAi-based GMPs have been designed to express either a double-stranded RNA (dsRNA) or an artificial microRNA (miRNA) precursor. These molecules are cleaved by Dicer/Dicer-like proteins into a pool of small RNAs that are 20–30 nucleotides long (small interfering RNAs [siRNAs] or miRNAs) and which specifically bind the target/messenger RNA (mRNA) with perfect or nearly perfect complementarity (Burand and Hunter, 2013; Koch and Kogel, 2014; Cagliari et al., 2019). siRNAs and miRNAs bind to an Argonaute protein forming the RNAi-induced silencing complex which, based on sequence homology, targets cognate RNAs. Current RNAi-based GMPs typically express a dsRNA that is designed to either downregulate a plant endogenous mRNA (e.g., to alter nutrient composition), or a gene in pests or pathogens that infest these plants, the so-called environmental RNAi (e.g., Ivashuta et al., 2015).

Small interfering RNAs and miRNAs may also trigger silencing of genes in the plant other than the intended targets (i.e., *off-targets*) giving rise to unintended phenotypes (Casacuberta et al., 2015).

## Efsa’s Risk Assessment Activities on RNAi-Based GMPs

The European Food Safety Authority has undertaken several activities on the risk assessment of RNAi-based GMPs to define in which areas existing risk assessment approaches for GMPs are suitable, or require complementary or alternative strategies. These include:

1. *International scientific workshop “Risk assessment considerations for RNAi-based GM plants” (4–5 June 2014, Brussels, Belgium:* At this workshop, experts from academia, risk assessment bodies, non-governmental organizations, the European Commission and the private sector identified scientific uncertainties on the level of exposure of humans, animals and the environment to dsRNA/artificial miRNA and derived small RNAs, hereafter referred to as silencing RNAs, and as well as limitations of *in silico* methods to unequivocally identify potential off-targets (European Food Safety Authority [EFSA], 2014).
2. *External scientific reports:* EFSA commissioned three external scientific reports in which relevant scientific literature was reviewed systematically to further inform the molecular characterisation, FF safety assessment and environmental risk assessment of RNAi-based GMPs, and address issues identified in the workshop. The report supporting the molecular characterisation addressed dsRNA and miRNA pathways in different species, including mammals, arthropods and plants (Pačes et al., 2017), while the FF safety report focused on the kinetics and possible effects of non-coding (nc) RNAs, including silencing RNAs, and upon ingestion by humans and animals (Dávalos et al., 2019). The report in support of the environmental risk assessment considered environmental RNAi-related aspects in arthropods, nematodes, and annelids and molluscs (Christiaens et al., 2018).
3. *Internal note on the strategy for the prediction and risk assessment of off-targets*: In 2017, EFSA’s GMO Panel published an internal note^1^ on the strategy to identify/predict off-targets and risk assess their potential impact in RNAi-based GMPs. It built on the available scientific knowledge and is expected to evolve with the progress of the knowledge in the field.
4. *GMO Panel opinions of RNAi-based GMPs*: EFSA’s GMO Panel assessed market registration applications for the import and processing for food and feed uses of potato EH92-527-1 (including cultivation in the EU) and soybeans MON87705, 305423, MON87705 × MON89788, and 305423 × 40−3−2 (excluding cultivation) designed to downregulate plant endogenous transcripts that modulate amylose and starch content in potato tubers or fatty acid profile in soybeans (EFSA Panel on Genetically Modified Organisms [EFSA GMO], 2006a, 2012, 2015; EFSA Panel on Genetically Modified Organisms [EFSA GMO], 2013, 2016, respectively). More recently, the GMO Panel also assessed the maize events MON87411 and MON87427 × MON89034 × MIR162 × MON87411 that constitute cases of environmental RNAi (EFSA Panel on Genetically Modified Organisms [EFSA GMO], Naegeli et al., 2018, 2019, respectively). Maize MON87411 expresses, among others, an insecticidal DvSnf7 dsRNA that downregulates the *Snf7* transcript in the western corn rootworm (*Diabrotica* spp.), and confers protection against this major maize pest. Some aspects of the risk assessment of maize MON87411 are further discussed below.

A complete overview of EFSA’s activities is provided in Table 1.

**TABLE 1:** Overview of the activities of the European Food Safety Authority on the risk assessment of plants genetically modified with RNA interference.

EFSA activity	Topic	References
Scientific workshop	Risk assessment considerations for RNAi-based GMPs plants	European Food Safety Authority [EFSA], 2014
External reports	Literature review of baseline information to support the risk assessment of RNAi−based GMPs	Pačes et al., 2017
	Literature review of baseline information on ncRNA to support the risk assessment of ncRNA−based GMPsfor food and feed	Dávalos et al., 2019
	Literature review of baseline information on RNAi to support the environmental risk assessment of RNAi−based GM plants	Christiaens et al., 2018
GMO Panel Note	Internal note on the strategy for the identification/prediction and risk assessment of off-target silencing effects in plants	Annex II of the Minutes of the 118th GMO Panel plenary meeting (2017)^a^
GMO Panel scientific opinions	Assessment of potato EH92-527-1	EFSA Panel on Genetically Modified Organisms [EFSA GMO], 2006a,b
	Assessment of soybean 305423	EFSA Panel on Genetically Modified Organisms [EFSA GMO], 2013
	Assessment of soybean 305423 × 40-3-2	EFSA Panel on Genetically Modified Organisms [EFSA GMO], 2016
	Assessment of soybean MON87705	EFSA Panel on Genetically Modified Organisms [EFSA GMO], 2012
	Assessment of soybean MON87705 × MON89788	EFSA Panel on Genetically Modified Organisms [EFSA GMO], 2015
	Assessment of maize MON87411	EFSA Panel on Genetically Modified Organisms [EFSA GMO], Naegeli et al., 2018
	Assessment of maize MON87427 × MON89034 × MIR162 × MON87411	EFSA Panel on Genetically Modified Organisms [EFSA GMO], Naegeli et al., 2019

*GMP, genetically modified plant; ncRNA, non-coding RNA; RNAi, RNA interference. ^*a*^Available at: https://www.efsa.europa.eu/sites/default/files/event/171025-m.pdf (last accessed 02/03/2020).*

## Risk Assessment Considerations for RNAi-Based Plants

### Molecular Characterisation

RNA interference specificity is based on the sequence identity between small silencing RNAs and mRNA targets; however, other transcripts with sufficient sequence identity to the small silencing RNAs can also be targeted for destruction leading to off-target effects (European Food Safety Authority [EFSA], 2014; Federal Insecticide, Fungicide and Rodenticide Act [FIFRA], Scientific Advisory Panel [SAP], 2014; Ramon et al., 2014; Casacuberta et al., 2015). Thus, identifying off-targets would facilitate risk assessment. Off-targets could occur in the GMP itself, or in other organisms that are exposed to the GMP and derived products through consumption. Based on the available knowledge, EFSA’s GMO Panel (see text footnote 1) considers that for plants a group of *in silico* parameters enables the prediction of off-targets, while for human and animals the available tools may not allow for sufficiently reliable predictions (Pinzón et al., 2017). Bioinformatic analyses for off-targets is based on several criteria (e.g., degree and position of base-pairing between the small RNA and transcript) that determine the efficiency of silencing (reviewed by Pačes et al., 2017). Therefore, *in silico* target prediction algorithms are designed based on criteria related to the biochemical and thermodynamical properties of base pairing, among other filtering parameters (Rhoades et al., 2002; Pasquinelli, 2012). In addition, other factors that can impact these interactions and lead to off-targets, is the abundance of each small RNA produced (Pačes et al., 2017). Depending on whether a dsRNA or artificial miRNA is used, a heterogeneous pool of siRNAs versus a more homogeneous pool of miRNAs will be produced, impacting the silencing of the potential off-target gene (Pačes et al., 2017).

Based on the above, the GMO Panel developed a bioinformatics-based strategy for the risk assessment of plant endogenous RNAi off-targets^1^. The parameters for identifying off-targets in plants are applicable to both siRNAs and miRNAs, and are based on a conservative approach, relying primarily on knowledge from miRNA-target specificity that accounts for complementarity mismatches between the small RNA and target gene (Liu et al., 2014). This strategy was implemented for the assessment of maize MON87411 and MON87427 × MON89034 × MIR162 × MON87411 (EFSA Panel on Genetically Modified Organisms [EFSA GMO], Naegeli et al., 2018, 2019, respectively). The outcome of the analysis did not identify off-targets that would require further safety assessment.

Nonetheless, bioinformatic searches for potential off-targets are subject to limitations (Pačes et al., 2017). Therefore, the outcome of plant off-target analyses must take the agronomic/phenotypic and compositional field-trial data gathered as part of GMP market application into account, as they are designed to identify intended and unintended changes in GMPs. On a case-by-case basis, if a potential plant off-target is identified, additional experimental data may be needed to investigate the predicted silencing effect at transcript level (see text footnote 1).

DvSnf7 dsRNA is expressed in the plant tissues of maize MON87411 and MON87427 × MON89034 × MIR162 × MON87411, and induces, upon consumption by the corn rootworm, RNAi leading to pest mortality. Typically, for the molecular characterisation of GMPs, expression of new constituents (usually newly expressed proteins) is demonstrated and risk assessed with regard to FF safety. In this respect, the levels of the DvSnf7 dsRNA, have been measured in different plant tissues of maize MON87411 (Urquhart et al., 2015). However, since it is likely that plant-Dicer proteins may process some of the DvSnf7 dsRNA into siRNAs, EFSA’s GMO Panel considers that “*the levels of dsRNA are not a good proxy for the levels of the active siRNAs present in plants”* (see Pačes et al., 2017; EFSA Panel on Genetically Modified Organisms [EFSA GMO], Naegeli et al., 2018, 2019).

### Food and Feed Safety Assessment

As supported by the external scientific report (Dávalos et al., 2019), ncRNAs, including silencing RNAs, are ubiquitous constituents of human and animal diet. Dietary silencing RNAs are known to be rapidly degraded soon after ingestion due to the conditions (e.g., pH) and enzymes present in the gastrointestinal tract lumen, and due to several barriers that exist at cellular (e.g., intestinal mucosa) and intracellular (e.g., lysosomal system) levels, preventing their systemic absorption. Therefore, the amount of dietary silencing RNAs absorbed after FF ingestion can be considered negligible in humans and animals (mammals, birds and fish), unless chemical modifications increasing their stability are introduced. The reported widespread presence, yet at low abundance, of exogenous RNAs in human and animal biological fluids, must therefore be viewed critically as it may be due to technical artefacts and contamination (Dávalos et al., 2019). Systemic effects of plant-derived silencing RNAs ingested orally have not been reliably established. In any case, the negligible absorption would further limit the possibility of silencing RNAs to reach a tissue or functional location in sufficient amounts and thus the possibility to exert any biological effect.

The above considerations were taken into account for the assessment of the DvSnf7 dsRNA expressed in maize MON87411 by EFSA’s GMO Panel. Given that the DvSnf7 dsRNA is not chemically modified to increase stability in the plant and/or increase cellular uptake in the gastrointestinal tract and systemic absorption following oral administration, EFSA’s GMO Panel concluded that the DvSnf7 dsRNA and its derived siRNAs are not able to exert any biological effects once ingested by humans and animals. Therefore, no animal studies were deemed necessary to support the FF safety assessment of maize MON87411 (EFSA Panel on Genetically Modified Organisms [EFSA GMO], Naegeli et al., 2018). Nonetheless, Petrick et al. (2016) tested the DvSnf7 dsRNA in a 28−day oral repeated−dose toxicity study in mice and identified no adverse effects in the tested conditions.

### Environmental Risk Assessment

A concern addressed for the environmental risk assessment of GMPs, including pest/pathogen-resistant dsRNA-expressing ones, for cultivation is their potential to cause harmful effects to valued non-target organisms (NTOs), especially arthropods, and the ecosystem services they contribute to (EFSA Panel on Genetically Modified Organisms [EFSA GMO], 2010; Taning et al., 2019). For harm to occur from dsRNA-expressing plants, NTOs must be susceptible to the dsRNA expressed by the plant and ingest it in sufficient concentrations (Christiaens et al., 2018). Exposure can occur when NTOs feed on living plant material, or consume other plant parts (e.g., pollen) or plant-fed herbivores, or are exposed through plant root exudates into soil or aquatic environments (Dubelman et al., 2014; Fischer et al., 2017; Parker and Sander, 2017; Romeis et al., 2019). Once the dsRNA is ingested by the NTO, it must resist degradation in the gut, and be uptaken in sufficient quantities to activate the NTO’s endogenous RNAi machinery. The latter can occur, either locally at the point of uptake (i.e., in cells lining the gut), or systemically if the NTO is able to trigger systemic RNAi (Ivashuta et al., 2015; Chan and Snow, 2017). A final condition is that the loss of the target transcript adversely affects the NTO (Bolognesi et al., 2012; Baum and Roberts, 2014). Conditions in the gastrointestinal tract of arthropods (e.g., nucleases, cellular surface receptors/membrane channels) generally do not apply to humans and food-producing animals, with the exception of crustaceans. Moreover, the efficiency of RNAi has been shown to vary greatly between different arthropod orders (Christiaens et al., 2018).

The NTO risk assessment requires consideration of the potential for off-target gene silencing (Lundgren and Duan, 2013; European Food Safety Authority [EFSA], 2014; Federal Insecticide, Fungicide and Rodenticide Act [FIFRA], Scientific Advisory Panel [SAP], 2014), especially for NTOs that are known to be susceptible to the dsRNA from the RNAi-based GMP and that are expected to be exposed to it. Bioinformatic analysis could identify which NTOs harbour genes that share some level of sequence homology with the target gene in the target pest/pathogen. Also, sequence complementarity between the derived siRNAs and NTO transcripts, would be indicative of potential RNAi activity in the NTO (Roberts et al., 2015; Devos et al., 2019a, b). Such data could thus be used to inform the NTO selection requiring further consideration in the risk assessment. If lack of minimum sequence homology for RNAi activity is reliably confirmed, then no further assessment may be needed (Roberts et al., 2015). However, currently, *in silico* predictions are subject to substantial limitations due to: (a) lack of sequence information for all NTOs; (b) differences between NTOs in how the RNAi machinery functions with regard to mismatches; and (c) scientific uncertainty on the exact rules governing interactions between siRNA-mRNA pairs (Ramon et al., 2014; Christiaens et al., 2018). More research on the RNAi mechanisms, design of efficient algorithms for reliable predictions and more suitable genome data for relevant NTOs will increase the usability of bioinformatic data for the assessment of off-target silencing in NTOs (Roberts et al., 2015; Christiaens et al., 2018; Devos et al., 2019a, b).

An alternative, yet complementary approach for the assessment off-targets in NTOs is to conduct laboratory bioassays with representative NTOs that are exposed to the dsRNA (Whyard et al., 2009; Bachman et al., 2013, 2016; Pan et al., 2017; Haller et al., 2019; Shang et al., 2019). Representative NTOs can include surrogate species that are selected based on their sensitivity to the dsRNA, reliability and relevance (Romeis et al., 2013). Typically, this involves phylogenetically close relatives, and species that are representative of valued taxa or functional groups that are most likely to be exposed to the dsRNA. This approach is appropriate for the assessment of RNAi effects on NTO fitness and performance, without the need for sequence information from the tested NTO. In the case of the DvSnf7 dsRNA, Bachman et al. (2013, 2016) observed no adverse effects with any of the NTOs tested at, or above, the maximum expected environmental concentration. In some cases, the timing and duration of exposure necessary to achieve the RNAi response may be uncertain, as may be the most sensitive endpoints to measure. Consequently, in some cases, and investigation of dose-dependent responses for siRNA targets may be needed (Federal Insecticide, Fungicide and Rodenticide Act [FIFRA], Scientific Advisory Panel [SAP], 2014; Roberts et al., 2015; Devos et al., 2019a, b).

An unresolved yet contentious point of debate is whether laboratory bioassays with plant material are useful to capture unknown complexities and variability in RNAi-based GMPs (Lundgren and Duan, 2013; Federal Insecticide, Fungicide and Rodenticide Act [FIFRA], Scientific Advisory Panel [SAP], 2014; Devos et al., 2016; Arpaia et al., 2017). Further evidence may be needed to investigate the usefulness and relevance of such bioassays for the assessment of unintended effects of RNAi-based GMPs for cultivation on NTOs, and what triggers their need (Devos et al., 2019a, b).

## Conclusion

EFSA has taken several actions to determine whether the existing risk assessment approaches for GMPs are appropriate for the risk assessment of RNAi-based GMPs or require complementary or alternative approaches. Moreover, EFSA has closely followed RNAi-related activities of other international risk assessment bodies and regulatory agencies (e.g., RNAi FIFRA Scientific Advisory Panel White Paper [Federal Insecticide, Fungicide and Rodenticide Act [FIFRA], Scientific Advisory Panel [SAP], 2014]). To our knowledge, at the international level, no dedicated guidelines have been developed for the risk assessment and regulation of RNAi-based GMPs, confirming that existing science-based risk assessment approaches for GMPs are generally considered suitable for RNAi-based GMPs. However, the following specificities have been identified for the risk assessment of RNAi-based GMPs:

• For the molecular characterisation, EFSA’s GMO Panel, along with other risk assessment bodies, considers that the identification/prediction of off-targets can be performed with a bioinformatics-based approach in plants, relying on conservative criteria, while for human and animals the available tools may not allow for sufficiently reliable predictions. Bioinformatic searches are subject to limitations and should thus be assessed in conjunction with the information derived from agronomic-phenotypic and compositional field-trials data. Furthermore, EFSA’s GMO Panel does not consider the dsRNA expression levels in the GMP relevant for the FF safety assessment since they are not representative of those of the active siRNAs in a plant.

• For the FF safety assessment, it is noted that dietary silencing RNAs are generally rapidly degraded shortly after ingestion, unless chemical modifications increasing their stability are introduced, and face several cellular and intracellular barriers to their absorption. Therefore, the amount of absorbed dietary silencing RNAs can be considered negligible in humans and animals and limits the possibility to reach a tissue or functional location in sufficient amounts to exert any biological effect. Based on this, EFSA’s GMO Panel considers that in general no dedicated animal studies on the safety of silencing RNAs are necessary.
• For the NTO risk assessment of pest/pathogen-resistant dsRNA-expressing GMPs for cultivation, it is agreed that bioinformatic analyses could identify NTOs that harbour genes with some level of sequence homology to the gene intended for silencing in the target pest/pathogen, and thus aid the selection of NTOs that require further consideration in the risk assessment (Devos et al., 2019a, b). However, at present, the presence of RNAi activity in NTOs cannot be reliably predicted in all representative NTOs through bioinformatic data. Therefore, this approach cannot be used as a stand-alone tool yet (Ramon et al., 2014; Roberts et al., 2015; Devos et al., 2019a, b). To make more reliable predictions, further research is needed to define the exact rules for small RNA-target matches, design suitable algorithms and increase knowledge on genomes and their expression, especially in non-model lines and other species (Ramon et al., 2014; Casacuberta et al., 2015). Overall, the tiered-based strategy for NTO risk assessment can be used as outlined in EFSA Panel on Genetically Modified Organisms [EFSA GMO] (2010) and Federal Insecticide, Fungicide and Rodenticide Act [FIFRA], Scientific Advisory Panel [SAP] (2014). Laboratory bioassays are considered appropriate to assess RNAi effects on NTO fitness and performance. However, exposure parameters, the most sensitive endpoints to measure, and dose-response relationships for siRNA targets may need to be established for NTOs that are susceptible to RNAi, on a case-by-case basis (Federal Insecticide, Fungicide and Rodenticide Act [FIFRA], Scientific Advisory Panel [SAP], 2014; Devos et al., 2019a, b).

## Author Contributions

NP conceived and took the lead in writing the manuscript. NP, YD, AL, and EW wrote sections of the manuscript. FÁ-A contributed tables and figures and formatted the manuscript. NP, YD, and EW provided critical feedback. All authors read and approved the submitted version.

## Conflict of Interest

The authors declare that the research was conducted in the absence of any commercial or financial relationships that could be construed as a potential conflict of interest.

## Footnotes

1 Available at: https://www.efsa.europa.eu/sites/default/files/event/171025-m.pdf (last accessed: 02/03/2020).
